# The cross-cultural process of adapting observational tools for pediatric pain assessment: the case of the Dental Discomfort Questionnaire

**DOI:** 10.1186/1756-0500-7-897

**Published:** 2014-12-11

**Authors:** Anelise Daher, Judith Versloot, Luciane Rezende Costa

**Affiliations:** Health Sciences Graduate Program, Federal University of Goias, Goiania, GO Brazil; Faculty of Dentistry, University of Toronto, Toronto, Canada; Division of Pediatric Dentistry, Faculty of Dentistry, Federal University of Goias, Goiania, GO Brazil; Faculdade de Odontologia, Primeira Avenida, Setor Universitario, Goiania, GO 74605-220 Brazil

**Keywords:** Methodology, Transcultural study, Assessment, pain, Pediatrics

## Abstract

**Background:**

A rigorous cross-cultural adaptation process of an existing instrument could be the best option for measuring health in different cultures, instead of developing a new tool, and prior to psychometric and validation testing. The Dental Discomfort Questionnaire (DDQ), a validated instrument for assessing toothache in young children, has not been cross-culturally adapted so far. This study aimed to explore the detailed phases of the cross-cultural adaptation process of a pain assessment tool, presenting the example of the DDQ Brazilian-Portuguese adapted version.

**Methods:**

The study design was based on the universalist approach, which consists of a sequential analysis to assess the relevant phases of a cross-cultural process before testing the measures of the instrument: conceptual, item, semantic, and operational equivalences. Systematic information was gathered from the literature, expert discussions, translations, and pre-testing through cognitive interviews with Brazilian population.

**Results:**

Detailed description of the three major phases for a cross-cultural adaptation process was given. Notes of the changes done in the structure of the presented instrument (DDQ) were specifically pointed out at each phase. Conceptual and item analyses showed that there are similarities in the DDQ construct between the original and Brazilian cultures that require minor modifications. Translations and back-translations allowed the development of the preliminary Brazilian-Portuguese version of the DDQ, which was tested and underwent other minor changes to improve its comprehensibility.

**Conclusions:**

Describing the phases was important to show how changes are made in a cross-cultural adaptation process of an instrument. This also could help researchers in adapting similar pediatric pain assessment tools to different cultures. A Brazilian-Portuguese version of the DDQ was presented.

**Electronic supplementary material:**

The online version of this article (doi:10.1186/1756-0500-7-897) contains supplementary material, which is available to authorized users.

## Background

Pain is a common experience in children starting in infancy [1]. Part of the definition of pain by the International Association for the Study of Pain (IASP) summarizes pain’s relief relevance for health professionals, mainly those caring for children: “The inability to communicate verbally does not negate the possibility that an individual is experiencing pain and is in need of appropriate pain-relieving treatment. Pain is always subjective. Each individual learns the application of the word through experiences related to injury in early life” [2].

Although self-report is considered to be the gold standard for assessing pain [3], immaturity in communication in young children could be a barrier to understanding their feelings or how they can express their pain [3, 4]; therefore validated observational tools are recommended as more reliable than self-reports for facilitating pain assessment in young children [5, 6]. To the best of our knowledge, the Dental Discomfort Questionnaire (DDQ) is the only published validated observational tool for assessing exclusively dental pain in children under the age of five [7]. The first version of the DDQ included 12 items reporting pain-related behaviors accordingly child’s oral habits to be completed by parents on behalf of their children; the final version of the DDQ, validated in the Netherlands, has 8 items and good psychometric properties [7].

The English-language version of the DDQ has been used in several studies in the literature [8–13], but it has never been cross-culturally adapted through a rigorous methodology for its application in other cultures. Because child health care is influenced by language and cultural issues [14], particularly for the assessment of pain in children [15], exploring the original DDQ across different cultures is important before its extensive use in different communities. Moreover, there is a lack of published studies regarding other dental pain assessment instruments, specifically for children less than 5 years of age. In Brazil, there is no validated instrument for dental pain assessment in preschool children. As recently happened for health quality of life instruments for Brazilian population, researches at this point has two alternatives: developing a new instrument or cross-cultural adapting and then validating an existing one [16]. Our hypothesis was that cross-cultural adapting the DDQ could possible discharge the need of the whole process of developing a new instrument.

This study aimed to present the detailed cross-cultural adaptation process of a pain assessment tool across two different cultures and languages, based on the universalist approach and using the example of the Dental Discomfort Questionnaire adapted for Brazilian Portuguese-speaking parents/children before the validation testing.

## Methods

### Study design

This study, approved by the Research Ethics Board of the Federal University of Goias (UFG), Brazil (#127/09), followed the Declaration of Helsinki’s principles. During each phase of the study, anonymity was guaranteed, and the participants (professionals, professors and children’s parents) were informed about the research and asked to sign an informed consent form explaining the risks and benefits of the specific phase and the entire study.

The study design was grounded in the universalist approach for cross-cultural adaptation [17–21]. In psychology, cross-cultural universalist methodology assumes that even though a construct could be based on a universal underlying process, the meaning of the items of an instrument have to be adjusted for each culture [22]. Although the 8-item DDQ is the model that was validated for the English language, complete 12-item DDQ concerning child’s oral habits (Additional file 1) was chosen to be adapted intending to explore the equivalences and relevance of all of the original items in the Brazilian population.

### Conceptual equivalence and item equivalence

The DDQ was first obtained from the author of the original version. Then, the DDQ conceptual equivalents for the Brazilian culture were investigated by reviewing the pertinent literature to understand the convergent and divergent points of conceptual equivalence for the questionnaire’s structure as well as dental pain perceptions in the target population. To improve the investigation of conceptual equivalence, the theme (dental pain perception) was extensively discussed by pediatric dentists experienced in caring for children in a focus group.

Item equivalence was assessed in the same focus group. Five Brazilian pediatric dentists (purposive sampling) invited to participate in the focus group discussed their perceptions about preschoolers’ dental pain [23] and about the relevance and acceptability of each DDQ item (Table 1). Participants were asked to comment on each item of the DDQ for its relevance for use in Brazil using the following responses: relevant; relevant, but needing minor modifications; little relevance; or not relevant. Two questions were added to complement the investigation of the importance and acceptability of the DDQ items for use in the Brazilian population: “Are the main behaviors related to toothache exhibited by Brazilian children included in this questionnaire?” and “Is there some relevant behavior about toothache that is not included in the DDQ?”. The discussion was audiotaped and verbatim transcribed to be analyzed.

**Table 1:** **Participants’ opinions about the relevance of the Dental Discomfort Questionnaire (DDQ) items in Brazilian culture**

DDQ questions/items	Relevance of items ^(a)^ as judged by each of the five participants ^(b)^
Does your child have toothache?	(1), (1), (1), (1), (1)
If sometimes or often is the: Toothache during meals?	(1), (1), (1), (1), (1)
If sometimes or often is the: Toothache during the day?	(1), (1), (1), (1), (1)
If sometimes or often is the: Toothache during the night?	(2), (1), (2), (2), (1)
Do you notice the toothache yourself?	(2), (1), (1), (1), (1)
Does your child indicate the toothache to you?	(1),(1), (1), (1), (1)
Biting things off with their back teeth instead of their front teeth?	(2), (2), (2), (2), (2)
Putting sweets away just after starting eating?	(1), (3), (1), (3), (1)
Starting to cry during meals?	(2), (1), (1), (1), (1)
Having problems with brushing upper teeth?	(1), (1), (2), (2), (1)
Having problems with brushing lower teeth?	(1), (1), (2), (2), (1)
Having problems chewing?	(1), (1), (1), (1), (2)
Chewing at one side?	(1), (1), (1), (2), (1)
Suddenly grabbing his/her cheek during eating?	(1), (1), (1), (1), (1)
Suddenly crying at night?	(1), (1), (1), (1), (1)

^(a)^The participants were asked to determine the relevance of each question/item as follows: (1) relevant, (2) relevant, but needing minor modifications, (3) little relevance, or (4) not relevant.

^(b)^Purposive sampling strategy: 5 pediatric dentists from public service and private practice, and who had varying experience caring for children (1, 6, 9, 20 and 22 years of pediatric dentistry).

The decision regarding whether to continue the DDQ adaptation process for use in Brazil was based on the positive outcomes for the conceptual and item equivalences [17]: (A) for conceptual outcomes - the construct is likely to be equally valid in both the original and target cultures; the importance of the domains varies between the two cultures; one or more of the domains used in the original instrument is not relevant to the concept of dental pain in the target culture; and the domains are different in the source and target cultures; (B) for item outcomes - the items can be used in the target version without modification; the items require minor modifications, but may be used, more or less, in their original form; replacement items must be used; and neither the existing nor the replacement items can be used.

### Semantic equivalence

Because the languages for the source instrument (English) and the target instrument (Brazilian-Portuguese) are not similar, translation and back-translation were required [20]. The semantic equivalence was assessed to verify the similarity of the meanings and applicability of the terms between the versions and was based on the four steps proposed by Guillemin, Bombardier, and Beaton (1993) [20]: Forward translation, back translation, consensus version after an expert panel, and back translation of the consensus version (Brazilian-Portuguese to English) to be evaluated by the author of the original DDQ.

In this study, the translations were performed by three independent translators who were native Brazilian-Portuguese speakers: a retired high school professor with a Bachelor’s degree in Arts and Languages (particularly English), a professional translator, and a bilingual health professional with clinical expertise in child dental care (the only one who was aware of the questionnaire’s intention and subject). Back-translations were also performed by three other translators living in Brazil who were native English speakers and fluent in the idioms and colloquial forms of the Brazilian Portuguese language but were uninformed about the intent of the DDQ.

The back translations were compared with the original DDQ to solve any divergence problems, to determine the need for the elimination of any inadequate information for the target population and, finally, to present a preliminary consensus version (to be back-translated to English and sent to the author of the original DDQ for evaluation) or to recommend another translation/back-translation process. The experts were a professor with experience in public health and epidemiological studies using questionnaires, two pediatric dentists with substantial clinical experience, and two professors with a large amount of expertise in cross-cultural adaptations of questionnaires.

### Operational equivalence

Operational equivalence was evaluated by applying the preliminary version of the Brazilian-Portuguese DDQ to 30 Brazilian caregivers of preschool children (convenience sample) attending the UFG dental clinic or university hospital and using cognitive interviews to assess the format and comprehensibility of the translated version [17, 24]. Cognitive interviews were performed by one trained interviewer (principal investigator) to identify possible problems with a pre-testing exploratory analysis. The interviewer first explained the goals of the questionnaire and asked the caregivers to sign a consent form. The respondents filled out the questionnaire individually, with constant monitoring by the interviewer, who attempted to answer questions and resolve misunderstandings following the probing method of cognitive interviewing [24]. All of the interviews were audiotaped for further analysis.

After discussing the problems detected in the format and the difficulties reported by the respondents, the interviewer, one professor of pediatric dentistry and the main author of the original DDQ created the final version of the cross-cultural adapted Brazilian-Portuguese DDQ.

## Results and discussion

### Conceptual equivalence and item equivalence

The DDQ items and concepts were considered to be equally valid for testing in the Brazilian population. The items were found acceptable for use with minor or no modifications needed, excepted translation; thereby encouraging the study of other types of equivalence [18].

The constructs employed in the development of the original DDQ were considered appropriate for use in the Brazilian culture. Toothache as reported by parents of Brazilian children was used for epidemiological purposes in the most recent national survey [25] and children’s oral habits during daily activities as related to the DDQ items (eating, brushing teeth and sleeping) were adequate as pain-related behaviors in Brazilian children [26, 27].

Out of five participants in the focus group two found that only one DDQ item (“Putting sweets away just after starting eating?”) was of little relevance because they argued whether parents could in fact relate this action to a toothache (Table 1). However, the item was kept for further analysis because this issue could only be answered by measurement equivalence that was tested in a subsequent study [28]. All of the participants considered all of behaviors included in the DDQ to be adequate and did not suggest the addition of any item.

Strategies for reviewing the literature and discussing items with dentists specialized in pediatric dental care were considered to be adequate for evaluating the relevance of the questionnaire in the target population, as described in the published guidelines [17, 19]. Members of the target population were not involved in this process because the concepts included in the questionnaire had already been tested in similar Brazilian populations as part of other questionnaires or surveys; therefore, a review of the literature provided sufficient exploration. Thus, the focus group methodology enabled important in-depth understanding of the relevance of dental pain as perceived by health professionals with extensive experience caring for the target population [29]. Another exploration of Brazilian pediatric dentists’ perceptions about dental pain in preschoolers showed the need of a validated instrument to systematically assess dental pain in this age group [23].

### Semantic equivalence

A consensus-based preliminary Brazilian-Portuguese version was created by an expert panel that performed this process extensively reviewing the words, terms and expressions of the back-translated versions. After examining the meanings of terms in the three translated versions, all of the items were changed from the original DDQ to this preliminary version (Table 2). Extrapolating the aims of semantic equivalence, the participants on the panel suggested replacing the circles (Additional file 1) for the response options with a table containing columns for each option that needed to be marked.

**Table 2:** **Items from the Dental Discomfort Questionnaire (DDQ) in the original version and the preliminary Brazilian version**

Toothache items from the original DDQ (Part 1)	Toothache items after modifications (Part 1)
1. Does your child have toothache?	1. Does the child have toothache?
If *sometimes* or *often* is the:	
a. Toothache during meals	1a. How often does the child have toothache during meals?
b. Toothache during day	1b. How often does the child have toothache during the day?
c. Toothache during the night	1c. How often does the child have toothache during the night?
2a. Do you notice the toothache yourself?	2a. Are you aware when the child has a toothache?
2b. Does your child indicate the toothache to you?	2b. Does the child show you when he/she has a toothache?
**Oral habit items from the original DDQ (Part 2)**	**Oral habit items modifications (Part 2)**
Is your child:	(The expression “Is your child” was excluded)
1. Biting things off with their back teeth instead of their front teeth?	1. Does the child bite with the back teeth rather than the front teeth?
2. Putting sweets away just after starting eating?	2. Does the child get rid of (spit out) sweets immediately after starting to eat them?
3. Starting to cry during meals?	3. Does the child begin to cry during meals?
4. a. Having problems with brushing upper teeth?	4. a. Does the child have difficulty brushing the upper teeth?
b. Having problems with brushing lower teeth?	b. Does the child have difficulty brushing the lower teeth?
5. a. Complaining about earache during eating?	5. a. Does the child complain of earache during meals?
b. Complaining about earache during the day?	b. Does the child complain of earache during the day?
c. Complaining about earache at night?	c. Does the child complain of earache while sleeping?
6. Having problems chewing?	6. Does the child have difficulty chewing?
7. Chewing at one side?	7. Does the child chew on one side only?
8. Suddenly grabbing his/her cheek during eating?	8. Does the child suddenly squeeze his/her cheek when eating?
9. Suddenly crying at night?	9. Does the child suddenly begin crying at night while sleeping?

Because some children in Brazil are accompanied by their caregivers (such as grandmothers) and not their parents, the term “the child” was chosen instead of “your child” as a more appropriate reference for the child. Moreover, the experts suggested repetition of the initial question “Is your child” for each item to improve understanding. None of the modified terms affected the concept of the original DDQ, but the questions could be more easily understood by the respondents.

There is a lack of standardization for the semantic equivalence phase, despite its relevance for the cross-cultural adaptation process [21]. The steps taken in this study to accomplish DDQ semantic equivalence [20] were also reported in another study on two general pain assessment scales [30]. Finally, the preliminary version of the DDQ adapted for the Brazilian population was approved by the original DDQ author for testing in the next phase.

### Operational equivalence

All invited caregivers (n = 26 mothers, n = 4 other relatives) participating in the cognitive interviews were categorized as having low socioeconomic status and filled out the questionnaire on behalf of their 1-5-year-old children. The probing method was chosen for cognitive interviewing to facilitate the interviews for respondents giving them the chance to do not feel depressed to fill out the questionnaire and to immediately resolve any questions [24, 31]. This sample size is reasonably large for detecting problems with an exploratory aim related to this cognitive interviewing approach [32].

Because the respondents found that the examples provided in the orientation part of the questionnaire (before the items) were somewhat confusing, researchers replaced them with two statements: “Please do not leave any question blank (no response)” and “Fill out the questionnaire by marking an “x” for the alternative that best matches your response for each of the questions”. In addition, the table format recommended by the expert panel was replaced by parentheses with a space to mark an “x” before each choice, which is a more commonly used format in Brazil [33]. Respondents also showed confusion when transitioning from Part 1 (toothache) to Part 2 (oral habits) because the numbering for the list in Part 2 was restarted. Thus, the numbered list was continued through that transition, with the first question of Part 2 numbered as 4. Regarding the individual items, only item #8 had to be modified: “Does the child suddenly squeeze his/her cheek when eating?”. The term “squeeze” was sometimes understood as “bite” and was replaced by another term based on one respondent’s suggestion: “suddenly lean a hand on his/her cheek when eating”.

There is a lack of specific guidelines for the cross-cultural adaptation of scales for pain assessment, even knowing the complexity of cultural influence on the assessment of a child’s pain [15]. Therefore, the final cross-cultural version of the DDQ adapted for the Brazilian-Portuguese language (Additional file 2) was obtained based on a rigorous adaption methodology proposed for health related quality of life instruments [17, 18, 20]. For validation purpose, measurement and functional equivalences of the present adapted version were thoroughly explored in another report [28]. Herein, we presented how was carried out the cross-cultural process of a pain tool to be used in different cultures, using the example of the first detailed description of the adapted Brazilian-Portuguese version of the DDQ.

## Conclusions

The cross-cultural adaption process of the Dental Discomfort Questionnaire was imperative before its final validation testing, because it enabled the researches to define DDQ as an instrument with universal concepts that could be used for Brazilian children after modifying its format and a few terms. The description of each phase of the cross-cultural adaptation is also relevant to inspire others to revise child pain assessment tools across different cultures.

## Electronic supplementary material

Additional file 1: **The Dental Discomfort Questionnaire (original version).** (PDF 217 KB)

Additional file 2: **The Dental Discomfort Questionnaire, Brazilian Portuguese adapted version.** (PDF 209 KB)
