# Supplementary material for: The cross-cultural process of adapting observational tools for pediatric pain assessment: the case of the Dental Discomfort Questionnaire
Source: BMC Res Notes. 2014 Dec 11;7:897. doi: 10.1186/1756-0500-7-897 (PMC4295577; doi:10.1186/1756-0500-7-897)
Supplement: Supplementary file 1 — Additional file 1: The Dental Discomfort Questionnaire (original version). (PDF 217 KB) [file 13104_2014_3401_MOESM1_ESM.pdf]

Dental Discomfort Questionnaire

Dear parent / caretaker,

We would like to ask you a few questions concerning your child's behaviour. We would also like to use some information from your child's dental record anonymously in this study. If you agree, would you be so kind to fill in the name of your child, the date of birth and your signature below.

Thank you for your cooperation.
Name of your child: ____ boy / girl*
Date of birth: ____
Signature: ____
***Delete as appropriate.**

<u>EXAMPLE</u>: How to fill in this questionnaire

Question

How often does your child eat sweets?

**Answer**	**Never**	**Sometimes**	**Often**
If your child <u>never</u> eats sweets, colour the first circle	●	O	O
If your child <u>sometimes</u> eats sweets, colour the second circle	O	●	O
If your child <u>often</u> eats sweets, colour the third circle	O	O	●

<u>Toothache</u>

	**Never**	**Sometimes**	**Often**	**Don't know**
1. Does your child have toothache?	O	O	O	O
If <u>sometimes</u> or <u>often</u> is the:				
a. Toothache during meals	O	O	O	
b. Toothache during the day	O	O	O	
c. Toothache during the night	O	O	O	

	**Yes**	**No**
2a. Do you notice the toothache yourself?	O	O
2b. Does your child indicate the toothache to you?	O	O

Oral habits

	Is your child:	Never	Sometimes	Often
1.	Biting things off with their back teeth instead of their front teeth?	O	O	O
2.	Putting sweets away just after starting eating?	O	O	O
3.	Starting to cry during meals?	O	O	O
4.	a. Having problems with brushing upper teeth?	O	O	O
	b. Having problems with brushing lower teeth?	O	O	O
5.	a. Complaining about earache during eating?	O	O	O
	b. Complaining about earache during the day?	O	O	O
	c. Complaining about earache at night?	O	O	O
6.	Having problems chewing?	O	O	O
7.	Chewing at one side?	O	O	O
8.	Suddenly grabbing his/her cheek during eating?	O	O	O
9.	Suddenly crying at night	O	O	O

Thank you for your cooperation!
