# Supplementary material for: The cross-cultural process of adapting observational tools for pediatric pain assessment: the case of the Dental Discomfort Questionnaire
Source: BMC Res Notes. 2014 Dec 11;7:897. doi: 10.1186/1756-0500-7-897 (PMC4295577; doi:10.1186/1756-0500-7-897)
Supplement: Supplementary file 2 — Additional file 2: The Dental Discomfort Questionnaire, Brazilian Portuguese adapted version. (PDF 209 KB) [file 13104_2014_3401_MOESM2_ESM.pdf]

Dental Discomfort Questionnaire, Brazilian Portuguese adapted version

(versão adaptada do Dental Discomfort Questionnaire para língua portuguesa do Brasil)

Prezados pais ou responsável,

Gostaríamos de fazer algumas perguntas em relação ao comportamento da criança. Se você concordar, por gentileza preencha o nome da criança e a data de nascimento, e assine abaixo marcando o seu parentesco com ela.

Nome da criança: ____

☐ Menino ☐ Menina

Data de nascimento: __________________

Parentesco ☐ Mãe ☐ Pai ☐ Outro: ______________________________

Assinatura: ____________________________________

- **Favor não deixar nenhuma pergunta do questionário em branco (sem resposta)**
- **Preencha o questionário marcando um "x" na alternativa que melhor responde cada uma das perguntas**

Dor de dente

1. A criança costuma ter dor de dente?
( ) Nunca ( ) Algumas vezes ( ) Muitas vezes ( ) Não sei

Se nessa primeira questão você respondeu "algumas vezes" ou "muitas vezes", com que freqüência a criança costuma ter dor de dente:

a. Durante as refeições?
( ) Nunca ( ) Algumas vezes ( ) Muitas vezes

b. Durante o dia?
( ) Nunca ( ) Algumas vezes ( ) Muitas vezes

c. Enquanto dorme?
( ) Nunca ( ) Algumas vezes ( ) Muitas vezes

2. Você percebe quando a criança está com dor de dente?
( ) Sim ( ) Não

3. A criança mostra para você que está com dor de dente?
( ) Sim ( ) Não

<u>Hábitos bucais</u>

4. A criança morde com os dentes de trás ao invés dos da frente?
( ) Nunca ( ) Algumas vezes ( ) Muitas vezes

5. A criança joga fora (cospe) os doces logo depois de começar a comer?
( ) Nunca ( ) Algumas vezes ( ) Muitas vezes

6. A criança começa a chorar durante as refeições?
( ) Nunca ( ) Algumas vezes ( ) Muitas vezes

7. A criança tem problema para escovar os dentes de cima?
( ) Nunca ( ) Algumas vezes ( ) Muitas vezes

8. A criança tem problema para escovar os dentes de baixo?
( ) Nunca ( ) Algumas vezes ( ) Muitas vezes

9. A criança reclama de dor de ouvido durante as refeições?
( ) Nunca ( ) Algumas vezes ( ) Muitas vezes

10. A criança reclama de dor de ouvido durante o dia?
( ) Nunca ( ) Algumas vezes ( ) Muitas vezes

11. A criança reclama de dor de ouvido enquanto dorme?
( ) Nunca ( ) Algumas vezes ( ) Muitas vezes

12. A criança tem problemas para mastigar?
( ) Nunca ( ) Algumas vezes ( ) Muitas vezes

13. A criança mastiga só de um lado?
( ) Nunca ( ) Algumas vezes ( ) Muitas vezes

14. A criança leva a mão na bochecha de repente enquanto come?
( ) Nunca ( ) Algumas vezes ( ) Muitas vezes

15. A criança começa a chorar de repente à noite enquanto dorme?
( ) Nunca ( ) Algumas vezes ( ) Muitas vezes

Muito obrigado!
